# The Role of Social Media in Communication and Learning at the Time of COVID-19 Lockdown—An Online Survey

**DOI:** 10.3390/dj11020048

**Published:** 2023-02-10

**Authors:** Mohammed Nahidh, Noor F. K. Al-Khawaja, Hala Mohammed Jasim, Gabriele Cervino, Marco Cicciù, Giuseppe Minervini

**Affiliations:** 1 Department of Orthodontics, College of Dentistry, University of Baghdad, Baghdad 1001, Iraq; 2 School of Dentistry, Department of Biomedical and Dental Sciences and Morphofunctional Imaging, University of Messina, Via Consolare Valeria, 1, 98125 Messina, Italy; 3 Multidisciplinary Department of Medical-Surgical and Dental Specialties, University of Campania “Luigi Vanvitelli”, Via Luigi De Crecchio 6, 80138 Naples, Italy

**Keywords:** COVID-19, social media, postgraduate, orthodontics

## Abstract

This study aimed to assess orthodontic postgraduate students’ use of social media during the COVID-19 lockdown. Ninety-four postgraduate students (67 master’s students and 27 doctoral students) were enrolled in the study and asked to fill in an online questionnaire by answering questions regarding their use of social media during the COVID-19 lockdown. The frequency distributions and percentages were calculated using SPSS software. The results showed that 99% of the students used social media. The most frequently used type of social media was Facebook, 94%, followed by YouTube, 78%, and Instagram, 65%, while Twitter and Linkedin were used less, and no one used Blogger. About 63% of the students used elements of social media to learn more about orthodontics staging, biomechanics, and various approaches in managing orthodontic cases. About 56% of students tried uploading and downloading scientific papers, lectures, movies, presentations, and e-books from social media, while communication with professionals and searches about orthodontic products were reported in 47% of students’ responses. On the other hand, 43% of the responses favored sharing orthodontic information and posts for teaching and discussion purposes. Generally, social media plays leading roles in the communication with, learning of, sharing of information with, and supervision of patients from a far during the COVID-19 lockdown.

## 1. Introduction

The Internet was initially introduced in 1982 as a means of communication between researchers; since 1994, the scope of its services has been broadened to benefit the whole community [1]. The Internet has since evolved continuously, and both general and healthcare-interested people have utilized it as a source of health-related knowledge in recent years. In 2011, searching for information related to health was considered the third most predominant online activity [2], and in 2012, interest in the medical field made up 72% of the online activity of mature internet users [3].

After that, Internet usage changed quickly, and the development of social media altered the mode by which individuals communicate socially via the Web. Social media can be defined as “online practices and technologies used by people to share insights, opinions, perspectives and experiences” and is important for diagnosis and therapy by dentists [4].

Websites such as Facebook, MySpace, and Twitter allow people to share information at any time about any subject. At the same time, blogs and wikis encourage ongoing knowledge sharing. Video websites such as YouTube offer a platform where humans can share their videos immediately with anybody in the world [5,6,7].

Promoters of the use of social media in the medical field suggest that these websites allow practitioners to communicate and connect with patients. Fisher et al. [8] confirmed this through a study in which 83% of the patients communicated via social media, and 56% of those patients asked their healthcare professionals to communicate with them through social media. Furthermore, a systematic review performed on the present literature considering social media in healthcare found that 72% of the examined studies advocated the usage of social media communication tools in healthcare [9].

Dental field researchers recommended that marketing via social media is the dental marketing future, as well as has an influence on patient behaviors and habits [6]. Marketing through social media is a cost-effective way to find thousands of new patients who are looking for a practitioner’s expertise [10,11]. Social media is also a necessary and robust reputation-making tool. Edwards et al. [12] found that the first factor in choosing an orthodontist was an excellent reputation. Furthermore, according to recent research concerning the anticipated quality of treatment among healthcare workers, reputation (66%) scored higher than both professionals’ recommendation (40%) and treatment cost (28%) [13].

Patients can rate their satisfaction with a healthcare provider online. However, these scores do not provide the necessary details regarding the actual experiences of the patients. Patients can also use social media to deliver their experiences to a more attentive and broader audience via different messaging techniques. In addition, social media websites can be utilized to share positive patient experiences and to respond to negative ones [14]. Although using social media to obtain healthcare linked information has been illustrated in the literature [15,16,17], its use specifically in the field of orthodontics is limited [18].

At the end of 2019, specifically in December, coronavirus disease 2019 (COVID-19) was discovered as an acute respiratory infectious disease in Wuhan, China. Later on, on 11 February 2020, the World Health Organization (WHO) proclaimed COVID-19 a pandemic with a high mortality rate and morbidity [19,20]. This virus can be spread chiefly by direct contact with and airborne droplets from individuals who have been infected and even asymptomatic carriers, with a higher degree of transmission in crowded places such as universities, schools, and supermarkets [21,22].

The Internet is increasingly used to seek out clinical and theoretical information as a result of growing technical breakthroughs [23,24]. Online continuous professional development (CPD) activities have grown in popularity as a result of this [25]. Furthermore, dependence on online activities has intensified as a result of the COVID-19 pandemic and the subsequent lockdowns [26]. Electronic e-learning is a well-established practice in orthodontics at the institutional level for undergraduate and postgraduate education [27], but it was only sometimes used before the COVID-19 pandemic to hold international conferences and CPD activities [26,28]. Webinars are well-established e-learning techniques in the field of medical education [29]. They are virtual learning sessions based on information and communications technology. Several platforms upon which to conduct these online sessions are currently available, and their delivery and attendance are facilitated by the use of computers and mobile devices. The benefits of remote learning—cost effectiveness, and time and location flexibility—are all provided by being a virtual activity [30].

Globalization, technological advancements, competition for e-learning options, and decreased public financing have all had a significant impact on higher education [31]. Three models are included in e-learning: In the adjunct model, online learning supplements face-to-face learning; in the blended model, face-to-face learning is integrated with online learning; and in the pure online model, all learning content is delivered through technology without any in-person instruction, giving students the greatest degree of independence. In the pure online paradigm, learning can be individual or collaborative, and it can be provided synchronously (virtually or face-to-face) or asynchronously (text-based Internet) [32,33,34].

Since orthodontic treatment is lengthy and needs restricted oral hygiene measures and instructions, patients may have faced some problems during the pandemic.

The null hypothesis is that orthodontic students have not used or gained benefits from social media during the COVID-19 pandemic; the aims of the current study were to evaluate the elements of social media most preferred by orthodontic postgraduate students and to inspect the prospective advantage of social media in learning, marketing, and the communication of policies about orthodontic practices during a COVID-19 lockdown.

## 2. Materials and Methods

After gaining approval from the ethical and scientific committees (no. 183 in 28 April 2020), this study was conducted on postgraduate students in the Department of Orthodontics, College of Dentistry/Universities of Baghdad, Mosul, Hawler, and Sulimania.

This study did not quantify the sample size since it sought to recruit as many postgraduate residents as possible over the designated time frame, so ninety-four postgraduate students were enrolled in the study to participate in filling an online questionnaire on Google Forms about their use of social media during the period from May to July 2020. The form consisted of the following questions:

- E-mail:
- Age:
- Gender: Male, Female
- Type of study: M.Sc., PhD.
- Did you use social media?
  Yes, which type?
  o Facebook
  o Youtube
  o Instagram
  o Twitter
  o Linkedin
  o Blogger
  No, Reasons for not using social media:
  o Time wasting
  o Privacy may be violated
  o I do not have time
  o Ethical issues
  o Not beneficial
  o Patients may post unfair remarks
- Why do you use social media?
  o To communicate with my friends and family
  o To communicate with my patients
  o To communicate with other professionals
  o To search for orthodontic products
  o To learn more about orthodontics regarding staging, biomechanics and different modalities for treatment
  o To upload and download scientific papers, lectures, movies, presentations and e-books
  o To share orthodontic information and posts for teaching and discussion purposes

The collected responses were analyzed in the SPSS program (version 25) to obtain the frequency distributions and percentages.

## 3. Results

**Figure 1:**
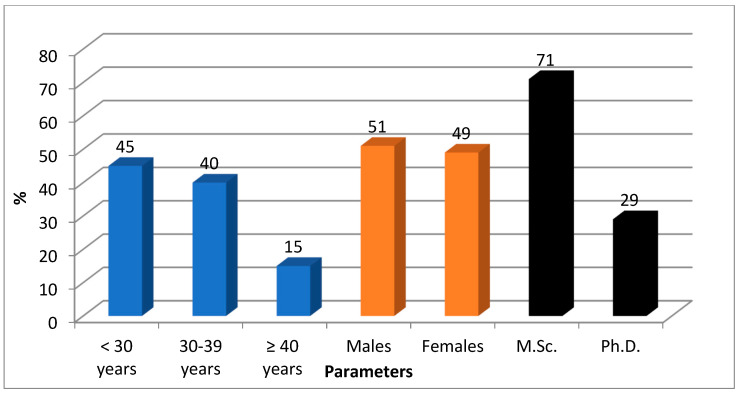
The demographical data of the participants.

The demographical data of the participants are presented in Figure 1. Ninety-four students participated; of them, 67 (71%) were master’s students, and 27 (29%) were doctoral students.

Regarding age, 42 (45%) were less than 30 years, 38 (40%) were between 30 and 39, and only 14 (15%) were 40 years and older. According to the gender distribution, 48 (51%) were males and 46 (49%) were females.

Complete information about the answers of the participants is shown in Table 1. From the whole sample, only one student did not use social media because he had no time, while 93 (99%) students used social media.

The type of social media most frequently used by the participants was Facebook (94%), followed by YouTube (78%) and Instagram (65%), while Twitter and Linkedin were used less (9 and 10%, respectively), and no one used Blogger.

Regarding the reasons for using social media (Table 2), 89% of responses reported communication with friends and family. About 63% of the students’ replies were regarding learning more about orthodontics staging, biomechanics, and various approaches to managing orthodontic cases.

One of the options for using different social media was uploading and downloading scientific papers, lectures, movies, presentations and e-books; this option was reportedly the reason for use in 56% of the answers.

Communication with the professionals and searching about orthodontic products were reported in 52% of the answers, while 43% favored sharing orthodontic information and posts for teaching and discussion purposes.

## 4. Discussion

When in-person schooling must be discontinued, distance learning is an alternative that might offer further educational opportunities. The idea of distance learning was first developed in the USA in the 18th century, facilitated by the mail service [35] and interactive telecommunication networks that linked students, resources, and instructors [36]. According to Garrison [37], there were three technological eras that contributed to the growth of distance learning: letters, telecommunications, and computers. Online education is increasingly popular in today’s culture as a means of facilitating effective instruction without requiring students to physically assemble [38]. The existing literature has already thoroughly investigated a number of benefits of distance learning using apps. Online education, a common kind of distance education, has been shown by Ebuete et al. [39] to allow teachers and students to successful teach and learn with the use of digital apps [40]. Additionally, uninterrupted teaching and learning can continue with online education [41,42]. Since remote learning is not constrained by physical boundaries and is available in other nations as well, it is more likely to make the best use of available resources [43,44]. Another area of distance education that is crucial in times of crisis is emergency remote education (ERE) [45]. ERE is characterized as a brief switch from a traditional classroom setting to an online one in response to an unexpected emergency [46]. ERE is different from distance learning in that it offers temporary teaching assistance in a quickly constructed manner and is only consistently provided during emergencies or crises [47].

The preamble of social media has modernized the mode by which people socially intermingle via the Web [1]. Originally, social media networks were made for personal use and then used successfully by businesses of all sizes to promote their products or services and to communicate with current and prospective consumers [48].

Social media has taken a momentous task in health care in recent times, as reported in several studies [16,17,18,19]. Social media marketing offers many advantages over traditional advertising as it is a collaborative, profitable, and more efficient solution used to promote services and products, notably as customers spend most of their time online [49,50,51]. During the COVID-19 pandemic, social media and Google services provided several advantages by making it simple to communicate with coworkers virtually and to obtain a wealth of information.

Daily lifestyles and activities have been changed unexpectedly with the emergence of COVID-19, as has, of most importance, communication with patients, friends, professionals, and students in delivering the required scientific materials. In current reviews performed by Izzetti et al. [52] and Ather et al. [53], the findings showed that both qualified dentists and dental students are most likely to become infected when they are in direct contact with patients as part of their academic learning and practice so the majority took leaves from work due to the COVID-19 pandemic’s consequences on their social and professional lives, notably dentists (orthodontists) who were working during the initial wave. Prior to the COVID-19 pandemic, the working lives of orthodontists were typical. However, due to the COVID-19 pandemic, some orthodontists only accepted emergency cases, while others did not because of the risk of becoming infected by the disease and other viral co-infection [54]. Because they quit working, some orthodontists suffered psychological breakdowns. On the other hand, this period gave orthodontists a lot of time to read some books and scientific papers, to watch a lot of online lectures and presentations, and to spend time with their families. This is according to Hasan et al. [55], who studied how the COVID-19 pandemic affected the behavior and reactions of a sample of Iraqi and Turkish orthodontists. Bsoul et al. [56] stated that dental care professionals should be well prepared to resume their practice in the most challenging circumstances and should take all the protective measures to protect themselves [57].

In teledentistry, which is a branch of telehealth, patients’ clinical information is exchanged and treatment planning is carried out over long distances using a variety of telecommunication techniques, including imaging [58]. This approach can help lower the risk of viral transmission and can increase adherence to social segregation policies. During the pandemic, 74.7% of orthodontists reported that they followed up with their patients using teledentistry or virtual consultations using smartphone apps [59]. Rahman et al. [60], in their study using questionnaires answered by 52 patients, stated that patient experiences with the usage of teledentistry were positive, evidenced by claims of patient satisfaction with the usefulness, dependability, and convenience of use. Orthodontists should think about modifying patient paths and employing teledentistry procedures as a consultation tool when planning the recovery of services in light of social distancing and lockdown measures.

Millions of students were forced to continue their education via virtual platforms that facilitated the completion of lessons via distance learning due to the extreme contagiousness of the virus, which made it hard to continue routine daily activities, including carrying out and attending lectures in person [61].

As a result, students were forced into situations of social isolation due to quarantines required to adhere to the rules of social distancing and to prevent the spread of the virus. This could have affected their study habits, particularly in those who frequented libraries and study rooms [62].

Undoubtedly, university students in faculties such as medicine and dentistry, whose training is focused on attending (in hospitals and/or classrooms) practical internships critical to their future careers, are particularly affected [63].

The situation had a significant impact on medical and dental students because of the stress associated with worrying that they might contract the virus and spread it to others as well as worrying that they might not have had enough time to prepare properly due to interruptions in teaching [64].

In addition to negatively impacting physical health, the COVID-19 pandemic has been the major cause of widespread anxiety and mental disease since January 2020 [65]. According to the Encyclopedia of Psychology, anxiety is characterized by tense sensations, anxious thoughts, and bodily changes such as elevated blood pressure. In addition, health anxiety is distinguished by uncomfortable feelings, physiological sensations, thoughts and pictures of danger, avoidance, and other protective actions [66]. The continual flow of information regarding the pandemic, reminders of mortality and unpredictability of health status, the implementation of lockdowns keeping people homebound, and uncertainty in financial stability have damaged the mental health of communities throughout the world [67]. Alhaj et al. conducted the first study on the mental health of neurosurgery residents and discovered that 90% of residents were impacted by the pandemic [68]. Shortage of personal protection equipment and extended work hours rendered the emotional and physical health of healthcare professionals even more challenging [69]. Knowledge and education on infectious illnesses may positively affect dentists’ viewpoints and attitudes while treating patients with infectious disorders; nevertheless, contradictory data have also been documented [70].

Postgraduate students were concerned about their limited exposure to operating procedures, retaining clinical competence, and endangering the health of their families, which ultimately led to burnout [71]. Moreover, using new technologies such as intra-oral scanners was highly recommended during this period [72].

Distance learning undoubtedly provides some benefits, including reduced travel time, the ability to learn in one’s own home, more flexibility, and a huge decrease in expenditures. On the other hand, there are undoubtably some drawbacks to distance learning, including an increase in distractions, issues with internet connectivity, an increase in anxiety and stress, and a lack of space and technological resources [73]. In a recent study performed by Sultan et al. [74], it has been found that COVID-19-related knowledge was clearly concomitant with anxiety experienced by the orthodontic postgraduate students during training throughout the pandemic. Awareness concerning infections led to more apprehension around working during the pandemic, arranging for postgraduate exams, and disquiets about its undesirable effect on the total quality of the training platform.

Ninety-four postgraduate orthodontic students (67 M.Sc. (71%) and 27 PhD (29%)) participated in the current study investigating the role of social media in contact with their supervisors, instructors, patients, and family members using a web-based (Google Forms) questionnaire, and students were given access to it by means of several social media platforms including Telegram, Messenger, Viber, etc.

Regarding the age of the participants, the majority of the students were less than 40 years old, with 45% being less than 30 years and 40% being between 30 and 39 years, while 15% were more than 40 years old. The number of males was slightly more than that of females, i.e., 51% males and 49% females in terms of gender distribution.

Regarding the use of social media, all of the students, except one, used social media with varying frequencies, being higher for Facebook (94%), followed by Youtube (78%), Instagram (65%), Twitter (9%), and Linkedin (10%), and no one used Blogger. Previous studies [75,76,77] showed that the most popular social media used is Facebook, followed by Youtube, which agrees with the present study’s findings.

The reasons why social media use differed among the students were that about 89% of the participants obtained the benefit of communicating with their friends and family members, while 41% used social media as a means of communication with their patients as it is challenging to monitor treatment progress or to instruct patients on how to resolve some problems related to trauma or injury from the archwires or ligature wires remotely. Al-Silwadi et al. [78] and Scribante et al. [79] significantly improved the knowledge and oral hygiene of orthodontic patients after delivering verbal and written information supplemented by audio–visual information via YouTube and Instagram. More concerns about wearing elastics, activating expanders [80], and managing oro-facial pain [81] are of the priority by direct contact with the patients during this period.

Zotti et al. [82] found that using WhatsApp had a beneficial and long-term outcome on stability and compliance, as contacting patients improves the regularity of wearing removable retainers and attending scheduled follow-ups.

Although PhD students completed their educational course, first-year master’s students still had lectures and presented their seminars using online sources using Youtube and Zoom. Moreover, many live webinars were presented during the COVID-19 lockdown, using live streams on either Facebook or Zoom. These webinars allow orthodontists, orthodontics students, or residents to remain in contact for updated and evidence-based information. In the present study, 56% of the participants used social media to upload and download scientific papers, lectures, movies, presentations, and e-books, while 43% of the students used social media to share orthodontic information and posts for teaching and discussion purposes.

During the COVID-19 pandemic, Zarzycka et al. [83] looked at the influences of a distance learning environment both within and outside of the virtual classroom on communication and teamwork, as well as the function of social media in this process. Their study’s findings showed that using Facebook more often for work reasons enhances student collaboration and communication in online courses. A high level of engagement on LinkedIn and Facebook is crucial for communicating with educators, supporting the findings of the present study. According to the engagement hypothesis, students’ collaborative and communication processes are significantly impacted by their active involvement in online courses and high ratings for the online technologies they utilize. This study adds to the body of knowledge on remote learning by expanding our understanding of distance learning in the middle of a pandemic through the engagement theory lens. For all individuals who actively participate in the educational process, it also has practical ramifications.

Al-Taweel et al. [84] evaluated various features of technology-based (TB) learning during the COVID-19 pandemic in an online-based survey performed on undergraduate dental students from different Iraqi universities. The students showed a low–moderate degree of satisfaction and a positive attitude towards TB learning and the quality of presentation of the scientific materials. Moreover, Herr et al. [85] found that South Korean dental students preferred and adapted well to web-based learning in pediatric dentistry. This may not be generalizable to postgraduate students as their expectation, practice, intelligence, and ability to manage (TB) learning is better than undergraduate students.

According to a study by Zheng et al. [86], dental students generally had positive opinions regarding online education during the COVID-19 pandemic, and their perceived interaction with professors and other students was a predictor of their willingness to take an online course. Most significantly, this is the first study in dentistry education to show that online learning during the pandemic may provide learning results that were comparable with or superior to those produced by face-to-face learning prior to the pandemic. The results of our study might make a substantial contribution to the body of knowledge on online education during the COVID-19 pandemic in health sciences. As we reimagine the future of online learning, the findings could help guide the future design of online learning.

A new study conducted by Mheissen et al. [87] to recognize the attendees- and host-related aspects that could optimize learning and uptake from virtual orthodontic learning sessions proved that the major interests of attendees were learning clinical orthodontic tips and evidence-based orthodontics, while learning new teaching styles was of minor significance; this is in accordance with the findings of the present study as 63% of the participants obtained benefits from social media in learning more about orthodontics regarding staging, biomechanics, and different modalities for treatment.

Italian dental and medical students’ perceptions of online teaching and training were recently assessed by Spirito et al. [88], finding that most Italian students considered distance learning to be appropriate during a pandemic, yet they thought that the best teaching process was still in-person teaching.

Finally, Li et al. [89] studied the effects of COVID-19 on distance learning using traditional and digital appliances in 60 developing countries, and their research revealed that the challenges of distance learning, with the use of multiple appliances, particularly digital ones, are especially relevant to the majority of emerging economies and even to some developed countries. As a result, a more comprehensive examination of this subject is required. Additionally, they acknowledge that certain kinds of distance learning, such as ERE and online distance learning, are particularly significant at different stages of a crisis based on their systematic literature review. In this way, based on regional particularities, distance education should be further recognized as an alternative means of education and investigated.

One of the limitations of this study is the inclusion of only orthodontic postgraduate students in the survey as it limits the ability of the study to be generalized to other specialties. More studies are needed to clarify the behavior of students after a pandemic using the same questions.

Additionally, further studies are needed to investigate the psychological impact of COVID-19 on orthodontists, residents, and patients.

Special apps should be developed to manage the many problems that patients may encounter in such cases, and these apps should facilitate contact with patients, explaining the many harmful effects and their management; in addition, orthodontists and residents in all fields of dentistry, from oral medicine to tooth restoration, should adapt well to e-learning and should benefit from the new technology by being able to obtain information easily using these new technologies [90,91].

## 5. Conclusions

Generally, social media plays significant roles in the communication with, learning of, sharing information with, and the supervision of patients from a far during the COVID-19 lockdown.

## Figures and Tables

**Table 1:** Frequency distributions and percentages of the participant’s responses regarding the use and the type of social media.

Parameters	*N*	%
Did you use social media		
Yes	93	99
No	1	1
Total	94	100
Type of media used		
Facebook	87	94
YouTube	73	78
Instagram	60	65
Twitter	8	9
Linkedin	9	10
Blogger	0	0

**Table 2:** Frequency distributions and percentages of the participant’s responses regarding the reasons for using social media.

Reasons for Using Social Media	*N*	%
To communicate with friends and family	83	89
To communicate with patients	38	41
To communicate with other professionals	48	52
To search for orthodontic products	44	47
To learn more about orthodontics regarding staging, biomechanics and different modalities for treatment	59	63
To upload and download scientific papers, lectures, movies, presentations and e-books	52	56
To share orthodontic information and posts for teaching and discussion purposes	40	43

## Data Availability

The raw data used to support the findings of this study are available from the corresponding author upon request.
